# Familiarity mediated by body size predicts intraspecific aggression in farming damselfishes

**DOI:** 10.1007/s00265-025-03636-x

**Published:** 2025-08-30

**Authors:** Catherine E. Sheppard, Lisa Boström-Einarsson, Dan A. Exton, Gareth J. Williams, Sally A. Keith

**Affiliations:** 1 Lancaster Environment Centre, Lancaster University, Bailrigg, Lancaster, LA1 4YQ UK; 2 Marine and Environmental Sciences Centre (MARE) & Aquatic Research Network (ARNET), Laboratório Marítimo da Guia, Faculdade de Ciências, Universidade de Lisboa, Cascais, 2750-374 Portugal; 3 Departamento de Biologia Animal, Faculdade de Ciências Universidade de Lisboa, Campo Grande, Lisbon, 1749-016 Portugal; 4 Operation Wallacea, Wallace House, Old Bolingbroke, Spilsby, PE23 4EX UK; 5 School of Ocean Sciences, Bangor University, Menai Bridge, LL59 5AB UK

**Keywords:** Neighbour-stranger discrimination, Intraspecific aggression, Territoriality, Familiarity hypothesis, Dear enemy

## Abstract

**Abstract:**

Aggressive territoriality can have significant benefits for resource acquisition yet is a costly behaviour. Selection should therefore favour mechanisms that allow individuals to modify their behaviour to maintain and defend their territory whilst minimising costs. One such mechanism between intraspecific territorial competitors is neighbour-stranger discrimination. The familiarity hypothesis suggests that territory holders will respond less aggressively to neighbours they recognise than to strangers they do not recognise. Conversely, in systems where neighbours pose a greater threat to territory loss than strangers, the threat-level hypothesis predicts that neighbours will elicit a greater aggressive response. We tested these opposing hypotheses in territorial farming damselfishes *Stegastes diencaeus* using a common bottle presentation experiment design to initiate aggressive responses by territory holders to neighbouring and non-neighbour individuals. Neighbours that were smaller in body size than the territory holder elicited the greatest aggressive response, whereas larger neighbours elicited the weakest. The aggressive response elicited by non-neighbours did not vary greatly with body size difference between the stimulus fish and territory holder. We demonstrate that aggressive response in territorial farming damselfishes is influenced by both familiarity and potential threat determined by body size. These findings add to the growing pool of evidence that an understanding of multiple factors is needed to identify the drivers of neighbour-stranger discrimination.

**Significance statement:**

Both familiarity and body size may mediate aggressive behaviour yet are not often included in the same study. Using manipulative field experiments, we investigated the interplay between familiarity and body size in shaping patterns of aggressive behaviour in farming damselfishes. We found that territory holders were less aggressive towards neighbours than non-neighbours, but only when they were larger than themselves. Our results showing an interaction between the effects of familiarity and body size on aggressive behaviour may hint at nuances in patterns of neighbour-stranger discrimination, such as dominance relationships.

**Supplementary Information:**

The online version contains supplementary material available at 10.1007/s00265-025-03636-x.

## Introduction

Territoriality is widespread across the animal kingdom yet carries significant cost in the form of aggression. Aggressive behaviour for the purpose of territory defence increases energy expenditure (Marler et al. 1995; Neat et al. 1998) and the risk of injury (Clutton-Brock and Huchard 2013). Thus, territory holders are expected to modify their behaviour in such a way that reduces these costs while still upholding their territory and minimising resource loss by intrusion. One such mechanism between intraspecific competitors is neighbour-stranger discrimination, where territory holders recognise and react differently towards neighbouring individuals than strangers (Ydenberg et al. 1988; Temeles 1994).

Territorial animals often demonstrate reduced aggression towards neighbours than strangers, termed the “dear enemy” effect. There are two alternative hypotheses that aim to explain this effect. The first, the familiarity hypothesis, predicts that frequent past encounters with the same individual will result in reduced conflict and aggression in subsequent encounters (Ydenberg et al. 1988; Temeles 1994). It is proposed that animals engage in fights to gain information about their opponent (Getty 1989). Neighbours have much less to learn about each other than strangers, resulting in fewer or reduced escalation of fights (Getty 1989). Similarly to familiarity, habituation to the same individual, such as neighbours who inevitably come into contact with each other more often, can decrease the intensity of subsequent encounters (Bee and Gerhardt 2001; Leiser 2003; Lehtonen and Wong 2017).

Alternatively, the threat-level hypothesis emphasises the importance of potential threat in determining aggressive response. When neighbours and strangers differ in their potential threat, the aggressive response of territory holders should be strongest towards the greatest threat (Temeles 1994). Established territory lines between individuals may lead to reduced conflict between neighbours, as observed in the root vole *Microtus oeconomus* (Rosell et al. 2008) and territorial cichlid fish *Neolamprologus pulcher* (Sogawa et al. 2016), conserving time and energy, whereas roving strangers seeking a territory of their own may be perceived as a greater threat to territory loss (Wilson 1975). Familiarity may also drive increased use of submissive behaviours (Hick et al. 2014).

Whilst the dear enemy effect has been widely documented (see Werba et al. 2022 for review), in some territorial species, neighbours elicit a stronger aggressive response than strangers, termed the “nasty neighbour” effect (e.g. Temeles 1990; Müller and Manser 2007; Newey et al. 2010; Munguía-Steyer et al. 2016). Typically, this effect is observed in systems where neighbours pose a greater threat than strangers. For example, intense competition may continue after territory boundaries are established if neighbours are continually trying to expand their territory (Müller and Manser 2007; Munguía-Steyer et al. 2016). The nasty neighbour effect may be more pronounced in species that hold multi-purpose, breeding territories, such as the Northern harrier *Circus cyaneus* (Temeles 1994), as territory holders stand to lose fitness as well as resources. Nasty neighbour effects may also be mediated by social status, as observed in crayfish *Procambarus clarkii*, where dominant females were preferred to fight with familiar subordinates (Tierney et al. 2013). It has also been suggested that social animals may exhibit nasty neighbour effects more often (Müller and Manser 2007; Newey et al. 2010), as strangers often represent smaller groups, and therefore a lower threat to territory takeover (Müller and Manser 2007).

A fundamental determinant of aggressive decisions, which is often overlooked in studies of neighbour-stranger discrimination, is differences in body size between opponents. Body size difference may mediate aggression by determining resource holding potential (RHP) (Green and Patek 2018) and social status (i.e. dominance). Larger individuals are expected to invest more in aggressive encounters than smaller individuals as they have a greater RHP and therefore a greater chance of winning a fight (Parker 1974). Social status is also often determined by differences in body size, as larger individuals with greater RHP gain dominance through past wins. As aggression is a costly behaviour, selection should favour aggression that is directed in such a way that maintains an individuals’ social status (Dehnen et al. 2022). Thus, it is expected that dominant individuals direct aggression to subordinates directly below them in the hierarchy (Dehnen et al. 2022). Both RHP and social status predict that individuals will display greater aggression towards smaller conspecifics (e.g. Green and Patek 2018; Tierney et al. 2013; Wright et al. 2019). Combining familiarity and body size differences in the same study will help to unearth potential nuances in neighbour-stranger discrimination.

Neighbour-stranger discrimination is observed in a variety of taxa, including birds (Temeles 1990; Godard 1993; Moser-Purdy et al. 2017), mammals (Müller and Manser 2007; Siracusa et al. 2017), invertebrates (Newey et al. 2010; Tierney et al. 2013; Munguía-Steyer et al. 2016) and fish (Leiser 2003; Lehtonen and Wong 2017; Sogawa and Kohda 2018). However, the mechanisms behind such discrimination have been inferred from studies limited in taxonomic breadth, with a disproportionate investment in birds, particularly breeding males (Werba et al. 2022). This imbalance limits our understanding of the generality of trends in neighbour-stranger discrimination across taxa (Werba et al. 2022). Marine species in particular have received very little attention, likely because of the challenges of underwater research. Moreover, the few studies testing the effect of familiarity on aggression in marine species have primarily worked in laboratory settings (e.g. Tricarico et al. 2011; Silveira et al. 2020). Though laboratory testing allows for greater manipulation and control and provides important insight into the drivers of such discrimination, investigating neighbour-stranger discrimination in the field, with the trade-off of smaller sample sizes, is critical to understanding how this phenomenon impacts processes in complex ecological systems.

Territorial farming damselfishes present an ideal model system to explore neighbour-stranger discrimination in the field. Individual farming damselfish of both sexes hold small contiguous territories which they aggressively defend from intra- and interspecific intruders. Encounters between neighbouring damselfishes along shared boundaries are frequent. Species of the genus *Stegastes* are some of the most aggressive (Ceccarelli et al. 2001) and hold multipurpose territories used for cultivating turf algae and, in the case of males, a space to care for and protect eggs. These species are highly site-attached (Itzkowitz et al. 1995; McDougall and Kramer 2007), taking occasional short forays (< 7 m; *Stegastes planifrons*; Itzkowitz 1978) outside of their territories. In *Stegastes diencaeus*, studies have found no evidence of nonterritorial roving individuals and infrequent relocation of territories by adults (McDougall and Kramer 2007) suggesting that individuals hold the same territory for long periods of time. This lack of relocation provides the opportunity for habituation and familiarity between neighbours.

We tested for the presence of dear enemy effects in the territorial farming damselfish species *Stegastes diencaeus*. In addition, we include differences in body size to investigate the interplay between familiarity and body size in mediating patterns of neighbour-stranger discrimination. We presented captured *S. diencaeus* neighbours and non-neighbours to territory holders and measured subsequent aggressive response of the free individual. This present study had two main objectives;

1. Determine whether *S. diencaeus* territory holders discriminate between neighbours and non-neighbours in their aggressive response (Do *S. diencaeus* exhibit dear enemy or nasty neighbour?).
2. Explore how body size differences between *S. diencaeus* territory holders and intruders mediates neighbour-stranger discrimination.

## Materials and methods

### Field methodology

We conducted our study between 01 June and 14 July 2023 at Coral View reef, Utila, Honduras (N 16.088233, W −86.910945). As our study involved focal animals in the field, it was not possible to record data blind. Eighteen focal *S. diencaeus* were chosen opportunistically (depth range 4.9–11.1 m), with the criteria of having at least one intraspecific neighbour with adjoining territory. The species were identified based on (1) dorsal and anal fins extending well beyond the base of the tail, and (2) an electric blue edge to the anal fin (differentiating them from other *Stegastes* species). Territories were marked with biodegradable flagging tape, which is a reliable method of identification (Snekser et al. 2009); Weimann et al. 2018) because this species is highly site-attached (McDougall and Kramer 2007). Previous work recorded a mean territory size of 0.55 m^2^ for *S. diencaeus* at this site (Sheppard et al. 2024) (maximum 1.19 m^2^; unpublished data). Therefore, focal *S. diencaeus* were located at least 5 m away from each other to maximise independence of the samples.

The sex of focal *S. diencaeus* could not be determined as there is no sexual dimorphism in this species. As both sexes hold territories for the purpose of farming algae, but only male territories are used for egg laying and guarding, it can be expected that sex affects aggressive behaviour relating to reproduction only, such as egg guarding or mating behaviour. No evidence of eggs or nests was observed, therefore differences between sexes in aggression should not affect our results substantially. Larval damselfish disperse on average around 50 km, with self-recruitment (ratio of larvae returning to their home reef compared with larvae from other reefs) averaging 15% (Hogan et al. 2012; Puebla et al. 2012). Given that genetic relatedness is unlikely to be significantly different between neighbours and non-neighbours, any effect of genetic relatedness on aggression was discounted.

## Bottle presentations

**Fig. 1:**
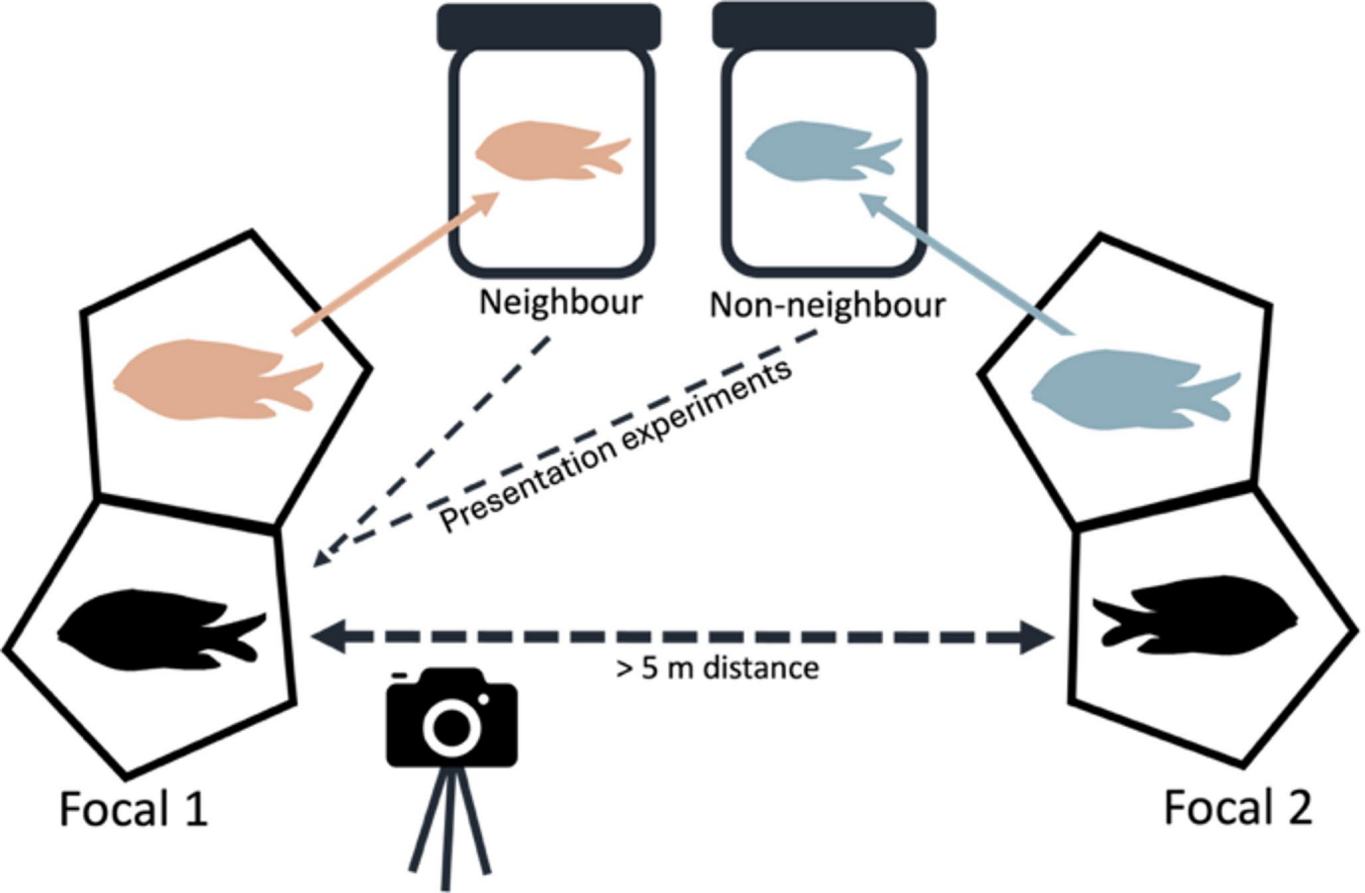
Bottle-presentation study design in respect to focal individual 1. Hexagons represent *S. diencaeus* territories. Black fish represent focal *S. diencaeus*, whilst orange and blue fish represent neighbouring *S. diencaeus*. Neighbouring *S. diencaeus* of two focal individuals (> 5 m apart), representing a neighbour and non-neighbour, were captured and contained in 3 L clear plastic bottles. After a period of acclimatisation, stimulus fish within their bottles were placed successively into the centre of the territory of the focal *S. diencaeus* and the aggressive response was video recorded for 3 min. The stimulus fish were presented to both focal individuals before being returned to their own territory

The aggressive response of *S. diencaeus* to intraspecific neighbours and non-neighbours was tested using a common presentation experiment (Harrington 1993; Haley and Müller 2002; Osório et al. 2006) which presents stimulus fish to focal individuals in clear bottles (Myrberg and Thresher 1974; Harrington 1993; Haley and Müller 2002; Osório et al. 2006; Fig. 1). We used 3 L clear plastic cylinders (14 × 24 cm) in which stimulus fish could swim freely, modified with mesh lids and perforated bottoms to allow waterflow. Teams of SCUBA divers captured two neighbouring *S. diencaeus*, one from each of two focal individuals, using barrier nets and spray bottles filled with a mix of ethanol and clove oil (3:1), a common fish anaesthetic (Whiteman and Côté 2002). Both captured *S. diencaeus* were used as stimulus fish for two focal individuals, offering a neighbour and non-neighbour stimulus, to reduce the number of animals required. Upon capture, stimulus fish within bottles were placed in their own territory, covered, and left to recover for at least 30 min. This also allowed any residual clove oil in the area to dissipate.

After the recovery period, stimulus fish within their bottles were placed successively into the centre of the territory of the focal *S. diencaeus* for 3 min, with the resultant behaviour recorded using a GoPro HERO camera (HERO Black 7|8|9) placed approximately 1 m away. Stimulus fish were presented from a different direction than their own territory. During presentations, SCUBA divers retreated to at least 2 m so as not to influence behaviour. This distance was deemed appropriate as *S. diencaeus* continue to display territorial behaviour in the presence of divers (per obvs), and teams had to be close enough on ethical grounds that they could observe the contained fish for signs of distress. The first ten focal *S. diencaeus* in our experiment were also presented with an empty bottle for 3 min to act as a control. As minimal response to the empty bottle was observed (see Results), control tests were discontinued, and subsequent focal individuals were presented with a neighbour and non-neighbour only. The order in which neighbours, non-neighbours and controls were presented was randomised using a random sequence generator and focal *S. diencaeus* were allowed to return to normal behaviour between presentations, confirmed by resumption of foraging or farming. Upon completion of the experiment, stimulus fish were returned and released back into their territories. During presentations to one focal *S. diencaeus*, a grouper, a known predator of damselfishes, intruded into the immediate area. This focal individual was removed from further analysis to exclude any effects of predator presence on aggressive behaviour. Our final dataset contained paired presentation experiments to 17 focal *S. diencaeus*. Due to the time restrictions of field experiments and SCUBA, each focal individual was tested once. The size of the presenting neighbour and non-neighbour was opportunistic.

## Behavioural analysis and size measurement

Behavioural videos were analysed using the BORIS software V. 8.6.2 (Friard and Gamba 2016). We recorded the total number of bites and rams directed towards the stimulus over the 3 min presentations. As it was difficult to discern when contact with the bottle was made, the number of bites and rams were summed to give a total count of aggressive displays. The standard lengths of focal and stimulus *S. diencaeus* were measured using ImageJ software (Schneider et al. 2012). Screengrabs were taken from behavioural footage such that the fish were positioned parallel to the bottle, allowing the bottle to be used for scale. Due to the restricted accuracy of this method, body size differences between stimulus and focal fish were recorded binomially as smaller (mean: − 1.0 cm, range: − 2.5 to − 0.1 cm; Fig. S1) or larger (mean = 0.7 cm, range = 0 to 1.9 cm; Fig. S1), with respect to the focal. Given that containing individuals undoubtedly changes their behaviour, and our ability to correctly identify behaviours (e.g. attack or defence), the behaviour of the stimulus fish was not measured. Whilst the behaviour of the stimulus fish may influence the behaviour of the territory holder, this was not possible to control but should be minimised by their restricted movement and lack of physical contact. Due to practical constraints and the close familiarity between observer and test subjects, observations were not blinded.

### Statistical analysis

All data manipulation and statistical analysis were conducted in R version 4.3.2 (R Core Team 2023). We ran Bayesian models using the brms package (Bürkner 2017) implemented in STAN (RStan 2023). We fitted total counts of aggressive displays against the type of stimulus (neighbour/non-neighbour) and body size difference (smaller/larger), with a Poisson distribution. The interaction effect between type of stimulus fish and body size difference was also included. We also fit total aggression against the type of stimulus fish and body size difference using subsets of data containing only larger or smaller conspecifics, and only neighbours and non-neighbours respectively (larger neighbours *n* = 6; smaller neighbours *n* = 11; larger non-neighbours *n* = 9; smaller non-neighbours *n* = 8). All models included focal damselfish ID as a grouping factor to account for individual variation in aggression. Weakly informative normal priors were used for all Bayesian models (Hadfield 2010). Models were run for 5000 iterations, with a warm-up of 1000 iterations over four chains. The adapt delta control parameter was increased to 0.95 or 0.99 to avoid any divergent transitions (Bürkner 2017). Model fit and convergence were visually validated using graphical posterior predictive checks, trace and density plots and Gelman-Ruban convergence diagnostic (R-hat) (Gelman and Rubin 1992). All models had an R-hat value of 1.00, signifying that the models had converged well. We tested our a priori hypotheses for our models using hypothesis testing. Hypothesis tests were two-way for comparing aggressive response to neighbours and non-neighbours (dear enemy or nasty neighbour) and one-way for comparing larger and smaller stimulus fish. For each test, we calculated the posterior probability (PP) and evidence ratios (ER). PP determines the probability to which our hypotheses were supported, and ER represents the extent to which the evidence supports our hypotheses compared with alternative hypotheses (Table 1). Note that two-way hypothesis testing tests whether two samples are the same, therefore posterior probability (PP) of below 95% indicates the samples are different.

**Table 1:** Summary of bayesian hypothesis tests. Hypotheses relate to total aggression by territory holder towards stimulus conspecific

Hypothesis	Median estimate (credible interval)	Posterior probability (PP)	Evidence ratio (ER)
Two-way hypothesis testing			
Neighbour = non-neighbour	0.36 (0.10–0.61)	0.74	2.8
Larger neighbour = larger non-neighbour	0.13 (−0.54–0.79)	0.98	40.25
Smaller neighbour = smaller non-neighbour	0.42 (0.15–0.69)	0.58	1.37
One-way hypothesis testing			
Smaller conspecific > larger conspecific	0.77 (0.45–1.1)	1.0	7999
Smaller neighbour > larger neighbour	1.93 (0.09–4.03)	0.96	22.77
Smaller non-neighbour > larger non-neighbour	1.04 (−0.95–3.02)	0.82	4.63

## Results

**Fig. 2:**
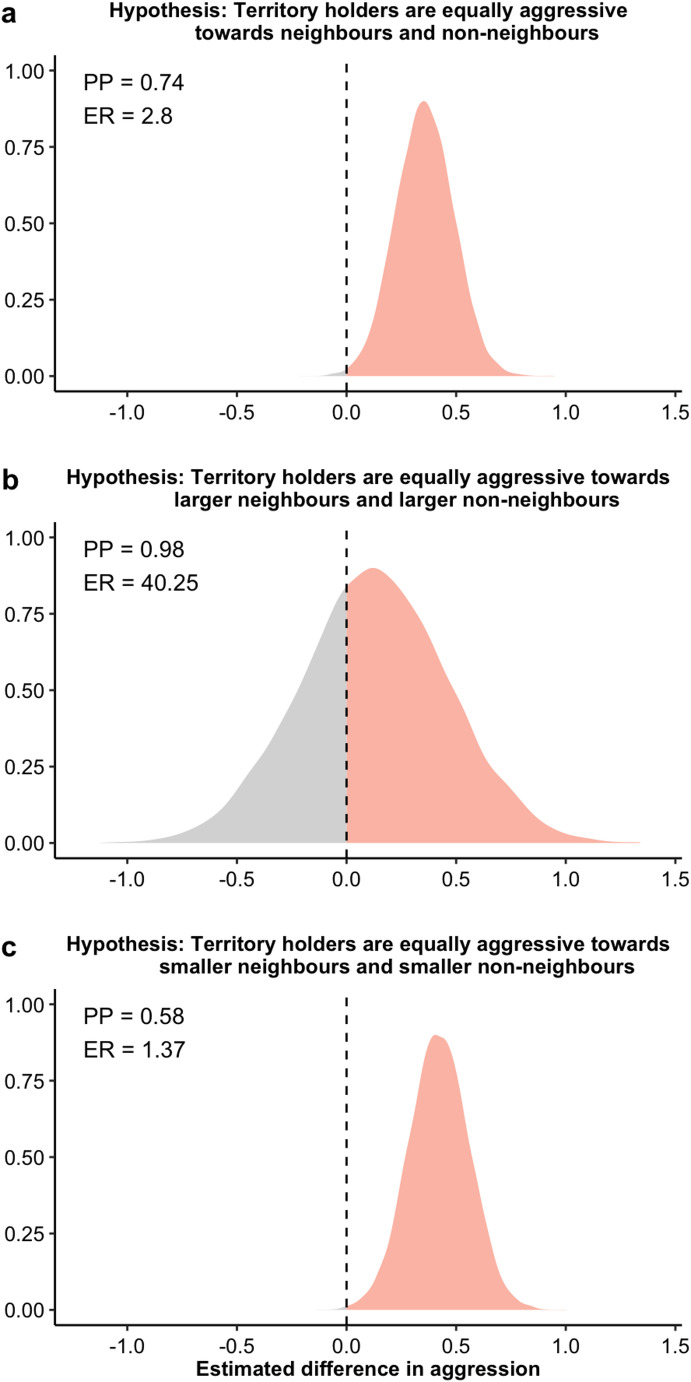
Bayesian posterior density plots of hypothesis testing, showing that (**a**) *S. diencaeus* territory holders are more aggressive towards neighbours than non-neighbours, (**b**) *S. diencaeus* territory holders are equally aggressive towards larger neighbours and larger non-neighbours, with body size being relative to themselves, and (**c**) *S. diencaeus* territory holders are more aggressive towards smaller neighbours than smaller non-neighbours. PP, ER and posterior density in orange represent the evidence to which *S. diencaeus* territory holders display greater total aggression

**Fig. 3:**
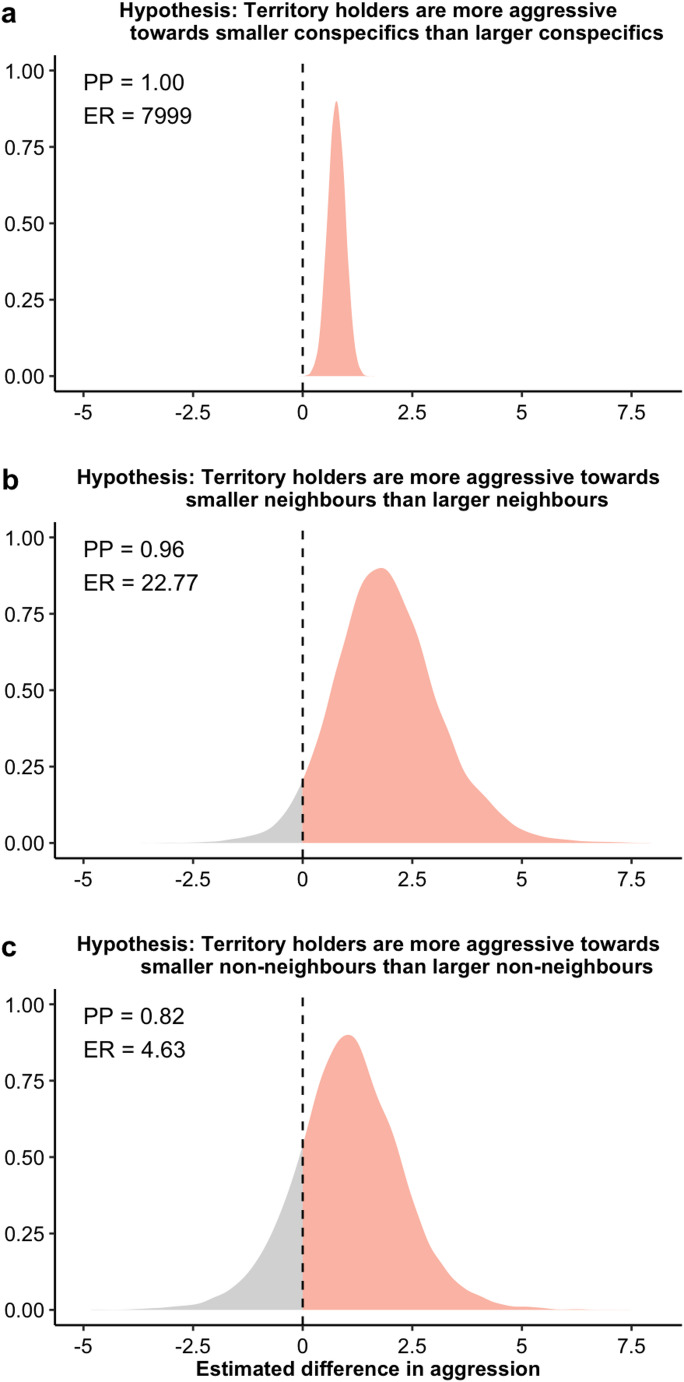
Bayesian posterior density plots of hypothesis testing, showing that *S. diencaeus* territory holders are (**a**) more aggressive towards smaller conspecifics than larger conspecifics, with body size being relative to themselves, (**b**) more aggressive towards smaller neighbours than larger neighbours, and (**c**) more aggressive towards smaller non-neighbours than larger non-neighbours. PP, ER and posterior density in orange represent the evidence to which *S. diencaeus* territory holders display greater total aggression

Hypothesis testing revealed that the total count of aggressive displays was mediated by both the type of stimulus fish and body size difference (Figs. 2 and 3; Table 1). Overall, there was strong evidence that *S. diencaeus* territory holders displayed greater aggression towards neighbours than non-neighbours (PP = 0.74, ER = 2.8; Fig. 2a). However, this trend was not consistent across body size difference. *S. diencaeus* territory holders were more aggressive towards smaller neighbours than smaller non-neighbours (PP = 0.58, ER = 1.37; Fig. 2c), but not towards larger neighbours than larger non-neighbours (PP = 0.98, ER = 40.25; Fig. 2b).

*S. diencaeus* territory holders displayed greater aggression towards smaller conspecifics relative to themselves than larger conspecifics (PP = 1.00, ER = 7999; Fig. 3a). This was consistent across neighbours (PP = 0.96, ER = 22.77; Fig. 3b) and non-neighbours (PP = 0.82, ER = 4.63; Fig. 3c). Extended hypothesis testing results are given in Table 1.

**Fig. 4:**
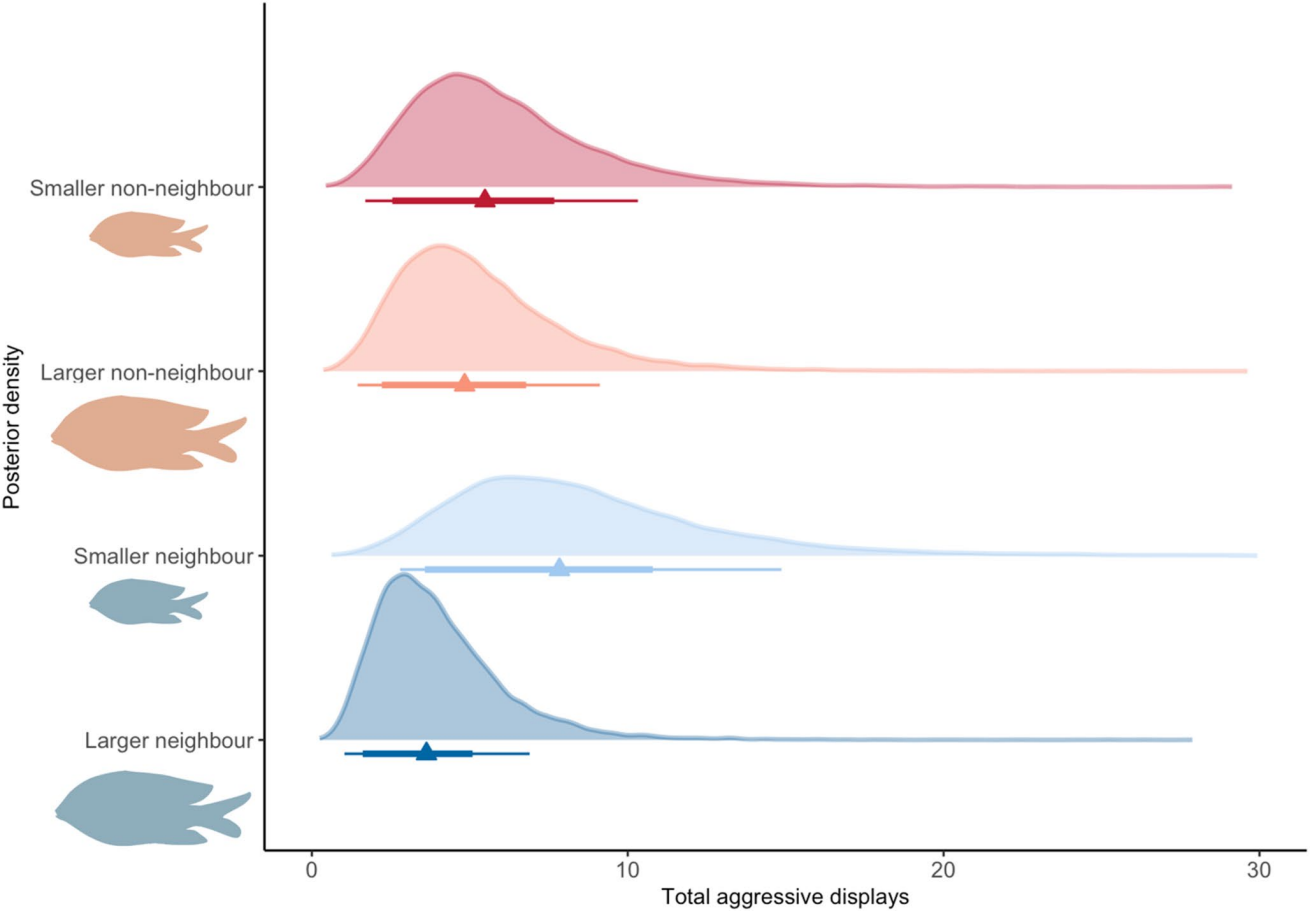
Total aggressive displays directed towards intraspecific stimulus fish by *S. diencaeus* territory holders is influenced by both familiarity and body size difference between territory holder and stimulus fish. Expected posterior predictions presented for *S. diencaeus* neighbours and non-neighbours. Point intervals represent median estimates and lines represent 90 and 70% highest posterior density intervals (HPDIs). Note x-axis limited to 30 for ease of viewing

Smaller neighbours elicited the greatest aggressive response (Median estimated posterior prediction = 7.97, 90% highest posterior density interval (HPDI) = 2.32 to 15.09; Fig. 4, S2; Table 2), whereas larger neighbours elicited the weakest aggressive response (Median estimated posterior prediction = 3.65, HPDI = 1.03 to 7.26; Fig. 4, S2; Table 2).

**Table 2:** Median estimates and 90% highest posterior density intervals of total aggression by territory holder

Stimulus type	Median estimate	90% Highest posterior density interval (HPDI)
Smaller neighbour	7.85	2.62–14.79
Larger neighbour	3.63	1.03–6.89
Smaller non-neighbour	5.48	1.61–10.26
Larger non-neighbour	4.84	2.54–9.44

Only one focal *S. diencaeus* was observed to aggressively ram the empty control bottle once, no other aggressive response was elicited by the empty control bottles. This demonstrates the lack of response to empty bottles, and that aggressive responses were elicited by stimulus fish.

## Discussion

The aggressive response elicited by intraspecific neighbours and non-neighbours in *S. diencaeus* territory holders was determined by both familiarity and body size difference between the territory holder and stimulus fish. Overall, aggressive response was highest towards neighbours, suggestive of nasty neighbour effects, yet the exact response was mediated by difference in body size. When looking at larger conspecifics only, *S. diencaeus* displayed similar levels of aggression towards larger neighbours as larger non-neighbours. We propose that the difference between level of threat posed by neighbours and non-neighbours, rather than familiarity, may offer a plausible explanation for our results.

The familiarity hypothesis suggests that familiarity between neighbouring individuals based on past encounters reduces aggressiveness of responses in subsequent interactions (Ydenberg et al. 1988). However, one key aspect that the familiarity hypothesis overlooks is that familiarity does not always confer potential threat (Temeles 1994). The threat-level hypothesis reasons that aggressive response should be strongest when directed towards the greatest threat (Temeles 1994). Typically, established territory lines between neighbours is expected to reduce conflict as neighbours are seen as a lower threat than roving strangers seeking their own territory (Temeles 1994; Rosell et al. 2008; Sogawa et al. 2016). However, in some territorial species, the opposite may be true. Neighbours may pose a greater threat to territory loss than strangers if neighbours are continually trying to expand their territory (Müller and Manser 2007; Munguía-Steyer et al. 2016). It has also been suggested that neighbours pose a greater threat when territories are used for reproductive purposes (Temeles 1990), or during the breeding season when mates are fertile (Moser-Purdy et al. 2017). In such circumstance, neighbours are predicted to elicit a greater aggressive response (e.g. Müller and Manser 2007; Newey et al. 2010; Munguía-Steyer et al. 2016). However, this assumes that all neighbours pose the same level of threat.

We found that the aggressive response by *S. diencaeus* to both neighbours and non-neighbours depended on the body size difference between opponents. One explanation for this may be that the body size of an intruder influences their potential threat in terms of RHP. Assessment of relative RHP relies on an individual’s assessment of their own RHP and that of their opponent and is expected to mediate the escalation of fights and the strength of aggressive response (Arnott and Elwood 2009). Larger individuals with greater RHP are expected to win fights, and therefore invest more into fighting than smaller individuals (Parker 1974). In terms of territorial species, this would suggest that territory holders respond more aggressively to smaller conspecifics. Indeed, we found evidence that *S. diencaeus* territory holders respond more aggressively to smaller conspecifics. This finding may suggest that *S. diencaeus* can assess the RHP and fighting ability of both familiar and unfamiliar individuals and adjust their behaviour accordingly.

Nasty neighbour effects alongside assessment of body size and associated RHP offers one plausible explanation for our findings. Nasty neighbour effects predict that neighbours elicit a greater aggressive response than strangers, whereas assessment of RHP predicts greater aggression is directed towards smaller conspecifics. Here, we found evidence that *S. diencaeus* respond more aggressively to neighbours than non-neighbours overall, but no such evidence was observed when looking at larger conspecifics only. *S. diencaeus* responded similarly to larger neighbours as larger non-neighbours, suggesting that size-mediated nasty neighbour effects may be present in this species.

An alternative explanation for our findings may be the presence of size-determined hierarchies between neighbours (Hemelrijk 2000; Hobson 2020; Tibbetts et al. 2022). Dominance hierarchies are widespread in group-living and aggregating species and are commonly established based on previous wins and losses during competitive encounters between neighbouring individuals (Tibbetts et al. 2022). Larger individuals with greater RHP are expected to win more fights and become dominants (Parker 1974), therefore dominance rank is often strongly correlated with body size (Favre et al. 2008; Clutton-Brock 2017; Wright et al. 2019). Typically, larger dominant individuals are expected to be most aggressive towards smaller subordinates (Tierney et al. 2013; Wright et al. 2019; Dehnen et al. 2022).

The application of these ideas to territorial farming damselfishes, such as *Stegastes*, has not been widely explored. Although farming damselfishes are described as solitary species, individual territories are often contiguous, forming intraspecific aggregations (Itzkowitz 1978; Robertson and Lassig 1980; McDougall and Kramer 2007). Though dominance hierarchies are typically present in group-living species, it has been argued that in territorial species, individuals with neighbouring territories may form dominant-subordinate relationships (rather than hierarchies) based on previous encounters (Rubenstein 1981; Fernö 1987). In *Stegastes partitus*, a territorial damselfish, dominance relationships have been found to be size-based (Sadovy 1985). Past examination of the spatial organisation of *Stegastes planifrons*, a closely related sister species of *S. diencaeus*, revealed that larger individuals, considered to be more dominant, held more preferable central territories than smaller individuals (Itzkowitz 1978). Following the expectation that dominant individuals direct greater aggression towards subordinates (Dehnen et al. 2022), we would expect territory holders to direct stronger aggressive responses towards neighbours that are smaller than themselves compared with those that are larger. We found strong evidence that *S. diencaeus* territory holders were more aggressive towards smaller neighbours, and therefore likely subordinate, than larger neighbours. Although this finding may suggest dominance relationships between neighbouring *S. diencaeus*, the aggressive response directed towards non-neighbouring *S. diencaeus* by territory holders was also associated with body size difference. Therefore, assessment of RHP between unfamiliar opponents may also be present.

An alternative perspective on the determinants of aggressive behaviour is to focus on the costs of aggressive encounters rather than the potential threat posed by an opponent. Costs of aggressive encounters are higher when the asymmetry in RHP between opponents is lower (i.e. opponents are more closely matched) (Arnott and Elwood 2009) and, intuitively, for individuals with lower RHP, such as those of smaller body size. Previous encounters with neighbours offer territory holders more information regarding their opponents RHP (fight to learn; Getty 1989), allowing for more accurate mutual assessment of the costs of subsequent interactions (Taylor and Elwood 2003; Arnott and Elwood 2009). In contrast, when faced with unfamiliar individuals, territory holders have less information on their opponent (Taylor and Elwood 2003; Arnott and Elwood 2009) and consequently, the costs are less predictable. Applying cost assessment theory, we might expect a weaker aggressive response (1) between unfamiliar individuals and (2) towards opponents with a larger RHP than the territory holder, as we observed in *S. diencaeus*. Assessment of the cost of an aggressive encounter may therefore mediate neighbour-stranger discrimination and provide an explanation to our findings.

Our findings rely on measuring aggressive response as the number of aggressive displays, in terms of bites and rams, directed by the territory holder to the stimulus fish. These behaviours are highly detectable to a human observer. However, damselfishes are known to communicate through a wide array of signals and displays which are more difficult to observe. For example, the presence and absence of UV patterns affects aggression between intraspecific *Pomacentrus amboinensis* (Siebeck 2004), and colouration patterns are integral to individual recognition in *Amphiprion hicinctus* (Fricke 1973) and *Stegastes planifrons* (Thresher 1979). Acoustic differences have also been shown to aid intruder discrimination between species (Weimann et al. 2018). The function of these more cryptic communications is largely unknown (however see Fricke 1973; Siebeck 2004) but there is potential for these signals to aid the assessment of a competitor’s RHP. Experiments to investigate the role of more cryptic signals in the communication of fighting ability and subsequent impact on aggressive territorial behaviour could add significant insight into these behaviours.

Aggressive behaviour associated with territoriality carries costs (Marler et al. 1995; Neat et al. 1998; Clutton-Brock and Huchard 2013). Natural selection should therefore favour any mechanism or strategy that allows a territory holder to minimise these costs whilst upholding their territory and preventing resource loss from intrusion (Arnott and Elwood 2009). We demonstrate that familiarity, interpreted with the added information provided by body size, predicts the level of aggressive response by *S. diencaeus* territory holders. Studies on neighbour-stranger discrimination are common (see Werba et al. 2022). However, our findings add to the growing pool of evidence that argue the need to include additional factors (e.g. dominance or body size: Tierney et al. 2013; Wright et al. 2019; density: Morales et al. 2014) when investigating the drivers of variation in territorial aggression between neighbours and non-neighbours. In doing so, we will be able to better predict not only individual behavioural responses but also how these responses scale up to influence population and community dynamics.

## Supplementary Information

Below is the link to the electronic supplementary material.

Supplementary Material 1

## Data Availability

All data and code associated with this study is available at https://github.com/cesheppard/damselfish_NSD. Raw videos available on request.
