# Supplementary material for: Familiarity mediated by body size predicts intraspecific aggression in farming damselfishes
Source: Behav Ecol Sociobiol. 2025 Aug 30;79(9):89. doi: 10.1007/s00265-025-03636-x (PMC12398450; doi:10.1007/s00265-025-03636-x)
Supplement: Supplementary file 1 — Supplementary Material 1 [file 265_2025_3636_MOESM1_ESM.docx]

# **Familiarity mediated by body size predicts intraspecific aggression in farming damselfishes**

Behavioral Ecology and Sociobiology

Catherine E. Sheppard^1,2^, Lisa Boström-Einarsson^1^, Dan A. Exton^3^, Gareth J. Williams^4^, Sally A. Keith^1^

^1^Lancaster Environment Centre, Lancaster University, Bailrigg, Lancaster, LA1 4YQ, UK

^2^Marine and Environmental Sciences Centre (MARE) & Aquatic Research Network (ARNET), Laboratório Marítimo da Guia, Faculdade de Ciências Universidade de Lisboa, Cascais, 2750-374, Portugal; Departamento de Biologia Animal, Faculdade de Ciências Universidade de Lisboa, Campo Grande, Lisbon, 1749-016, Portugal

^3^Operation Wallacea, Wallace House, Old Bolingbroke, Spilsby, PE23 4EX, UK

^4^School of Ocean Sciences, Bangor University, Menai Bridge, LL59 5AB, UK

Corresponding author: Catherine E Sheppard; [cesheppard@ciencias.ulisboa.pt](mailto:cesheppard@ciencias.ulisboa.pt)

**Supplementary Figures**


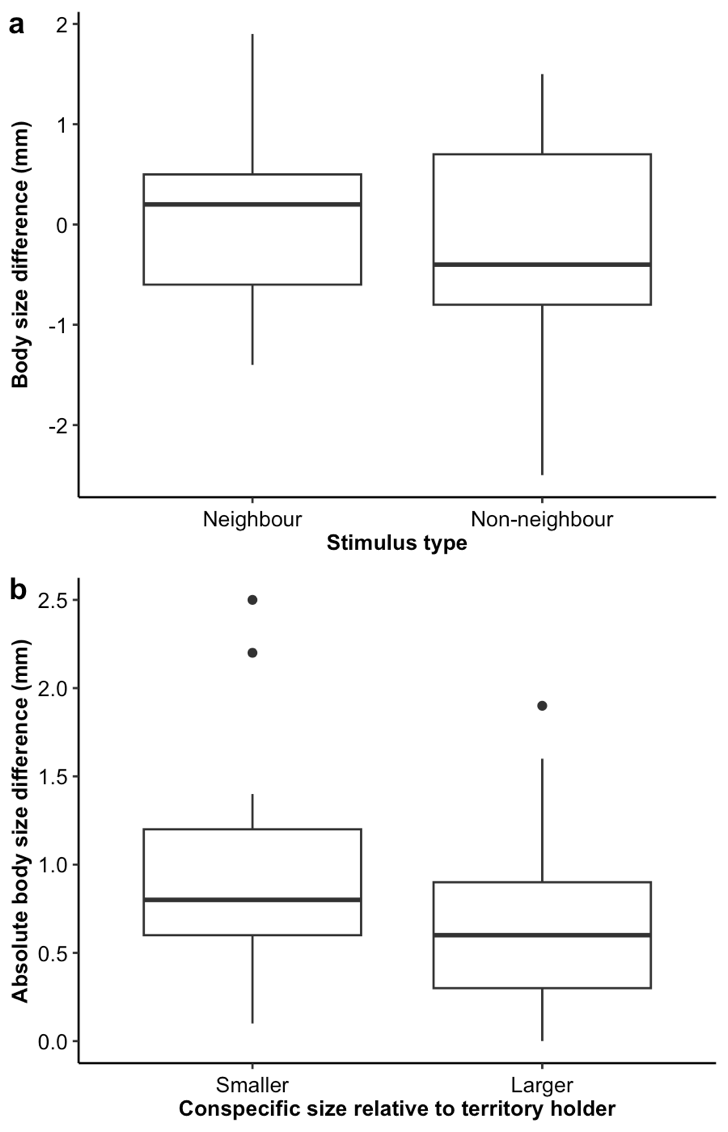


**Fig. S1** Boxplots of body size differences between stimulus fish and focal territory holder, showing separately a) the spread of body size differences of neighbours and non-neighbours, and b) the spread of body size differences of smaller and larger conspecifics. Boxplots represent medians, IQR and full extent of the data.


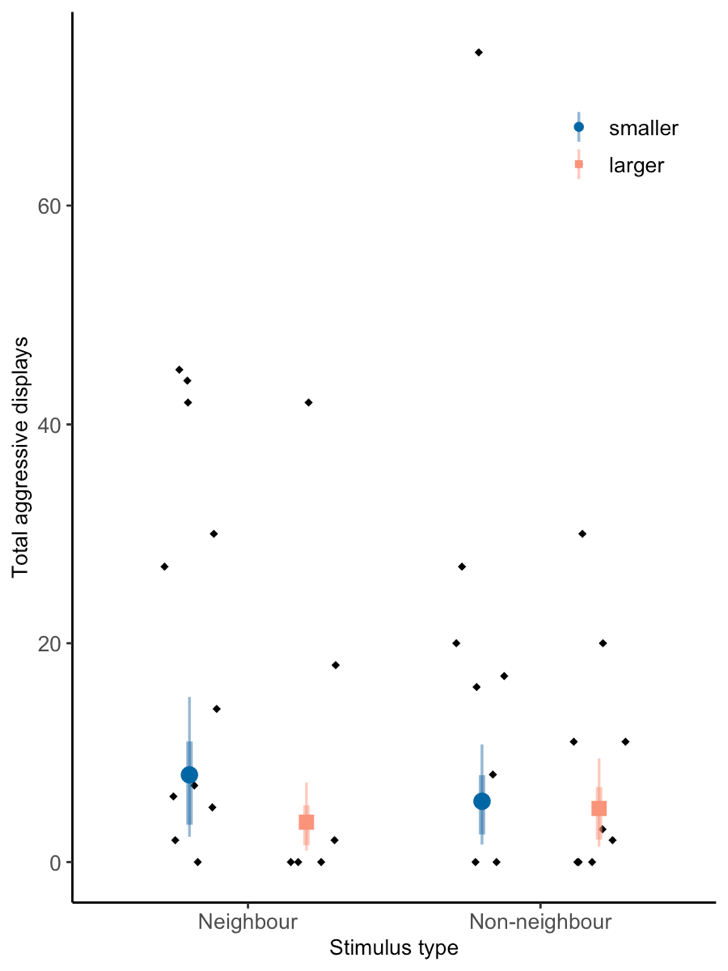


**Fig. S2** Total aggressive displays directed towards intraspecific stimulus fish by *S. diencaeus* territory holders is influenced by both familiarity and body size difference between territory holder and stimulus fish. Expected posterior predictions presented for *S. diencaeus* neighbours and non-neighbours. Point intervals represent median estimates and lines represent 90 and 70% highest posterior density intervals (HPDIs). Black points represent raw data
